# FAM20B Gain-of-Function Blocks the Synthesis of Glycosaminoglycan Chains of Proteoglycans and Inhibits Proliferation and Migration of Glioblastoma Cells

**DOI:** 10.3390/cells14100712

**Published:** 2025-05-14

**Authors:** Lydia Barré, Irfan Shaukat, Mohamed Ouzzine

**Affiliations:** UMR7365 CNRS-Université de Lorraine, Biopôle, Faculté de Médecine, CS 50184, 54505 Vandoeuvre-lès-Nancy Cedex, France; lydia.barre@univ-lorraine.fr (L.B.);

**Keywords:** FAM20B, proteoglycans, glycosaminoglycan synthesis, glioblastoma

## Abstract

Heparan sulfate (HS) and chondroitin sulfate (CS) proteoglycans (PGs) are essential regulators of many biological processes including cell differentiation, signalization, and proliferation. PGs interact mainly via their glycosaminoglycan (GAG) chains, with a large number of ligands including growth factors, enzymes, and extracellular matrix components, thereby modulating their biological activities. HSPGs and CSPGs share a common tetrasaccharide linker region, which undergoes modifications, particularly the phosphorylation of the xylose residue by the kinase FAM20B. Here, we demonstrated that *FAM20B* gain-of-function decreased, in a dose dependent manner, the synthesis of both CS- and HS-attached PGs. In addition, we showed that blockage of GAG chain synthesis by FAM20B was suppressed by the mutation of aspartic acid residues D289 and D309 of the catalytic domain. Interestingly, we bring evidence that, in contrast to FAM20B, expression of the 2-phosphoxylose phosphatase XYLP increases, in a dose dependent manner, GAG chain synthesis and rescues the blockage of GAG chains synthesis induced by FAM20B. In line with previous reports, we found that *FAM20B* loss-of-function reduced GAG chain synthesis. Finally, we found that FAM20B inhibited proliferation and migration of glioblastoma cells, thus revealing the critical role of GAG chains of PGs in glioblastoma cell tumorigenesis. This study revealed that both gain- and loss-of-function of *FAM20B* led to decreased GAG chain synthesis, therefore suggesting that a balance between phosphorylation and dephosphorylation of the xylose by FAM20B and XYLP, respectively, is probably an essential factor for the regulation of the rate of PG synthesis.

## 1. Introduction

Proteoglycans (PGs) are complex macromolecules that are found on the cell surface and in the extracellular matrix [1]. They are formed by the covalent attachment of one or more glycosaminoglycan (GAG) chains onto the core protein. PG–GAG chains interact with a large number of ligands including growth factors and morphogens and their receptors, as well as extracellular matrix structural molecules where they regulate activity and ligand stability [2,3]. PGs regulate several biological processes including cell–cell and cell–matrix interactions, development, signaling, proliferation, differentiation as well as various pathological conditions, such as inflammation, tumor progression, and viral and bacterial infection, [4,5,6,7]. Most commonly occurring PGs are chondroitin sulfate PGs (CSPGs) and heparan sulfate PGs (HSPGs). These PGs share a common tetrasaccharide linker region (GlcAβ1-3Galβ1-3Galβ1-4Xyl-*O*-Ser) for the attachment of the GAG chain onto the PG core protein. The first step in the synthesis of the tetrasaccharide primer is the addition of xylose on specific serine residues of the core protein. This initial and limiting step in GAG synthesis is catalyzed by xylosyltransferase I and II [8,9,10]. Subsequently, two galactose residues are attached to xylose by β1,4-galactosyltransferase 7 and β1,3-galactosyltransferase 6 (β4GALT7 and β3GALT6), and one glucuronic acid by β1,3-glucuronosyltransferase (GLCAT-I), therefore completing the synthesis of the tetrasaccharide primer. HS- and CS–GAG chains of PGs are built up on this linkage tetrasaccharide region by the alternate addition of *N*-acetylhexosamine and glucuronic acid residues [11]. The specificity of interaction between ligands and GAG chains is influenced by the fine structure of the GAG chain, primarily its sulfation pattern [12]. The tetrasaccharide primer also undergoes various modifications, including sulfation of galactose residues and phosphorylation of xylose [13,14].

*FAM20* (family of sequence similarity 20) contains three members *FAM20A*, *FAM20B*, and *FAM20C* [15]. Their function has recently been established [16,17]. The substrates for FAM20A are unknown; however, FAM20A is important for enamel biomineralization and tooth eruption [17,18]. Mutations in *FAM20A* are associated with various tooth disorders, named human amelogenesis imperfecta and gingival hyperplasia syndrome [19], and in some cases the renal calcification is also involved along with tooth disorders such as in enamel renal syndrome [18,20]. FAM20C is a Golgi casein kinase and is mainly expressed in the biomineralized tissues. FAM20C phosphorylates casein and the small integrin-binding ligand, N-linked glycoproteins (SIBLINGs), which are critical for biomineralization. Mutations in *FAM20C* are associated with Raine syndrome, characterized by a lethal osteosclerotic bone dysplasia [16,21,22]. Recently, FAM20C from the *C. elegans* (ce FAM20C) has been crystallized [23]. The 3D structure revealed the presence of a protein kinase-like fold with five disulfide and two asparagine residues for N-linked glycosylation. The ce FAM20C also contains a DFG (Asp-Phe-Gly) variant motif, in which the aspartate residue Asp^387^ co-ordinates the divalent cation required for catalysis and a catalytic segment that includes the putative catalytic aspartate residue, Asp^366^. The Asp^387^ and Asp^366^ residues are conserved in all the FAM20 family [23].

FAM20B is a kinase that phosphorylates the xylose in the tetrasaccharide linkage region of PGs [24,25]. Genetic studies showed that loss-of-function mutations in *FAM20B* in zebra fish decreases the amount of cellular GAG chains and causes cartilage and skeleton defects [26]. *FAM20B* knockout in mice results in embryonic lethality, and embryos showed multisystem organ hypoplasia and delayed development [17]. On the other hand, it has been shown that gain-of-function of XYLP, the 2-phosphoxylose phosphatase that de-phosphorylates xylose in the tetrasaccharide linker region, increases the amounts of GAGs in Hela cells and inversely, loss-of-function of XYLP decreases GAG synthesis [27].

The aim of the present study was to investigate the potential regulatory function of FAM20B in the synthesis of PG. Here, we demonstrate that *FAM20B* gain-of-function inhibits the synthesis of PGs and showed that both CS/DS– and HS–PGs are affected. Interestingly, we found that FAM20B inhibition of PG synthesis is dose-dependent and is rescued by the phosphatase XYLP. Furthermore, based on the crystal structure of ceFAM20C, we showed that aspartic acid residues D289 and D309 are essentials for FAM20B suppression of the synthesis of CS and HS-attached PGs.

## 2. Materials and Methods

### 2.1. Cell Lines and Culture Conditions

The Chinese hamster ovary cells (CHO-K1), CHO galactosyltransferase I deficient cell line pgsB-618, human embryonic kidney cells HEK293 (CRL-3216, ATCC, Manassas, VA, USA), human brain tumor T98G (CRL-1690, ATCC), human lung adenocarcinoma cells A549 (CCL-185, ATCC, Manassas, VA, USA) and human primary skin fibroblasts were cultured in DMEM (4.5 mg/mL glucose) or DMEM-F12 medium supplemented with 2 mM glutamin, 100 IU/mL penicillin, 100 µg/mL streptomycin and 10% fetal bovine serum (FBS, Dutscher, Bernolsheim, France) at 37 °C with humidified atmosphere in a 5% CO_2_.

### 2.2. Vector Constructions and Cell Transfection

*FAM20B* was amplified from human placenta cDNA library (Takara Bio, San Jose, CA, USA) by PCR using 5′GAATTCCACCATGAAGCTAAAGCAGCGAGTCGTG3′ (forward) including EcoRI site and 5′GGATCCTTACAAGTGTGAGAGAGCCATCCT3′ (reverse) primers including BamHI site, using Advantage GC 2 Polymerase Mix (Takara Bio). XYLP was cloned by PCR using 5′GAATTCCACCATGCT TTTCCGCAACCGCTTC3′ (forward) including EcoRI site and 5′CTCGAGTTAGAATCCTTCCCTGTGACATGC3′ (reverse) primers including XhoI site. The amplified *FAM20B* and *XYLP* products were ligated into pCR2.1-TOPO vector (Invitrogen, Carlsbad, CA, USA). Flag-XYLP and HA-XYLP cDNAs were generated by PCR. The coding region for *FAM20B* was excised and ligated into the pCMV vector (Stratagene, Valencia, CA, USA) by double digestion with EcoRI and BamHI to generate pCMV-FAM20B. The coding regions for Flag-XYLP and HA-XYLP were excised and ligated into the pCMV vector by double digestion with EcoRI and XhoI to generate pCMV-Flag-XYLP and pCMV-HA-XYLP. Decorin and HA-syndecan 4 cDNAs were generated by PCR and cloned into EcoRI and BamHI or EcoRI and XhoI sites of pCMV empty vector to generate pCMV-DCN and pCMV-HA-SDC4, respectively.

For transfection, cells were seeded in 6-well culture plate until 80% confluency and transfected with 250 ng of either pCMV-DCN or pCMV-HA-SDC4 in combination with 1 µg pCMV-FAM20B, pCMV-HA-XYLP, pCMV-Flag-XYLP, pCMV-FAM20B^D289A^, pCMV-FAM20B^D309A^ or pCMV-empty vector using lipofectamine 2000 transfection reagent (Invitrogen, Carlsbad, CA, USA) according to the manufacturer’s instructions. Expression of decorin and of syndecan 4 was analyzed at 48 h post-transfection by Western blotting.

### 2.3. FAM20B Knockdown

Sense 5′**CACC**GTATAGCCGAGACCATGTGG3′ and antisense 5′**AAAC**CCACA TGGTCTCGGCTATAC3′ oligonucleotides containing 20 bp sequence (underlined) targeting *FAM20B* exon 3 and cohesive ends (bold) with the vector were annealed and ligated into BbsI sites of pUC57-attbU6 sgRNA vector, which express gRNA using avian-derived U6 promoter. Cells were transfected with empty pUc57-attbU6 (control), pUc57 attbU6/FAM20BgRNA in association with pspCas9 plasmid expressing Cas9. To facilitate screening, cells were co-transfected with pSVneo plasmid which express the neomycin resistance. Positive cells were screened using resistance to neomycin and gene mutation was determined by sequence analysis. The *FAM20B* genomic region targeted was amplified by PCR using specific primers. Sequencing results indicated that *FAM20B* gene harbored frameshift mutation mediated by deletion of two nucleotides at the sites targeted by gRNA, creating premature stop codon that prevents the translation of full length FAM20B. All analyzed sequences from the transfected cell clones exhibited the same deletions in the *FAM20B* genomic region.

### 2.4. Site-Directed Mutagenesis

Site directed mutagenesis of the residues Asp289 to Ala289 and Asp309 to Ala309 in *FAM20B* were performed using the QuikChange XLII (Agilent, Santa Clara, CA, USA) according to the recommendations of the manufacturer. pCMV-FAM20B expression vector was used as template. Sense and antisense oligonucleotides introducing the desired mutations were for D289A: 5′CTGATTGGCAATGCTGCCCGCATCACTAT GAG3′ (forward) and 5′CTCATAGTGATGGCGGGCAGCATTGCCAATCAG3′ (reverse) and for D309A: 5′TGCTCATCCTTCTTGCTAATGCCAAAAGCTTTGG3′ (forward) and 5′C CAAAGCTTTTGGCATTAGCAAGAAGGATGAGC A3′ (reverse). Full length mutated cDNA FAM20B^D289A^ and FAM20B^D309A^ were checked by DNA sequencing.

### 2.5. N-Glycosylation Analysis

Cells were grown in a 6-well culture plate at 80% confluency then transfected with pCMV-FAM20B expression vector. At 24 h post-transfection the cells were lysed in the HEPES buffer, and protein concentration was measured by the Bradford method [28]. Twenty µg of protein was digested with PNGase F (New England Biolabs, Ipswich, MA, USA), which cleaves asparagine-linked (N-linked) oligosaccharides, according to the manufacturer’s instructions.

### 2.6. Western Blotting

Total protein from cells was extracted using RIPA buffer (150 mM NaCl, 50 mM Tris-HCl, pH 7.5, 1% deoxycholate, 0.1% SDS, 1% Triton X-100) supplemented with protease and phosphatase inhibitors (Roche Diagnostics, Indianapolis, IN, USA). Cell lysates were sonicated on ice, and protein concentration of the samples was determined by the Bradford method. Proteins from culture medium and from cell lysates (50 μg/lane) were separated on 10% SDS-PAGE gels, transferred to a PVDF membrane (Bio-RAD, Hercules, CA, USA), and subsequently blocked in PBS-Tween 20 containing 5% nonfat milk or 5% BSA. Membranes were then incubated overnight with primary antibodies directed against FAM20B (Cat# HPA 007409, 1/1000, Sigma-Aldrich, Saint Louis, MO, USA), decorin (Cat# MAB143, 1:1000, R&D Systems, Minneapolis, MN, USA), HA (Cat# 901501, 1:10000, BioLegend, San Diego, CA, USA), M2-Flag (Cat# F1804, Sigma-Aldrich) or β-actin (Cat#3700, 1/2000, CST, Danvers, MA, USA) followed by incubation with horseradish peroxidase-conjugated secondary antibodies (Cat# 7074, 1:2000, CST or Cat# 7076, 1:2000 CST, Danvers, MA, USA). Antibodies were diluted in 5% BSA/0.01% Tween 20 in PBS. The blots were then developed using Clarity Western ECL substrate (BIO-RAD, Hercules, CA, USA) according to the instructions of the manufacturer.

### 2.7. Metabolic Labelling of GAG Chains

Metabolic labelling of PG–GAG chains was carried out using ^35^S-sulfate incorporation method as described by De Vries et al. (1986) [29]. Briefly, cells were grown in a 6-well culture plate and transfected with pCMV-FAM20B, pCMV-FAM20B^D289A^, pCMV-FAM20B^D309A^, or pCMV empty vector. Cells were then radiolabeled with 10 µCi/mL of ^35^S-sulfate (Perkin Elmer, Wellesley, MA, USA) in sulfate free media containing 2% dialyzed FBS. After overnight incubation, the culture medium was collected, digested with papain (1 mg/mL), and ^35^S-labeled GAGs were precipitated by cetylpyridinium chloride (CPC) as described by Bronson et al. (1987) [30]. The CPC precipitated radiolabeled GAGs were separated by SDS-PAGE on a 4–12% Nu-PAGE gel. The gel was dried and exposed to autoradiography film. To measure the rate of sulfate incorporation into GAG chains of PGs, HEK293 transfected with pCMV empty vector or pCMV-FAM20B was radiolabeled with 10 µCi/mL of [^35^S]-sulfate for 6 h, then, conditioned culture medium was collected and digested with papain (1mg/mL). [^35^S]-labeled GAG chains were precipitated by CPC dissolved in solvable and mixed in scintillation fluid (Perkin Elmer, Wellesley, MA, USA). The radioactivity associated with GAGs was measured by liquid scintillation counting (Packard, Rungis, France).

### 2.8. Immunofluorescence Analysis

The CHO-K1 cells were grown on glass coverslips and transfected with FAM20B expression vector pCMV-FAM20B or with pCMV empty vector (control). At 36 h after transfection, cells were fixed with 3% (*w*/*v*) paraformaldehyde in PBS for 20 min and were permeabilized by treatment with 0.1% (*w*/*v*) Triton X-100/PBS solution for 4 min. After extensive washing in 0.2% (*w*/*v*) fish skin gelatin in PBS, cells were then incubated with primary antibodies anti- FAM20B (Cat# HPA 007409, 1/100 Sigma-Aldrich) and anti-HS (Cat# 370255-1, 1:100, AMSBIO, Abingdon, UK) for 20 min. Cells were washed several times in 0.2% (*w*/*v*) fish skin gelatin in PBS and incubated with secondary antibodies coupled with Alexa Fluor 555 and Alexa Fluor 488 (Cat# A-21428 or Cat# A-11017, Molecular Probes, Eugene, OR, USA) for 20 min. Cells were washed with PBS and nuclei were stained with Hoechst/PBS solutions then coverslips were mounted with Moviol (Calbiochem, San Diego, CA, USA) containing 1% propylgallate (Sigma-Aldrich). Digital images were captured with an inverted microscope—Lieca DMI3000 B (Leica Microsystems, Wetzlar, Germany).

### 2.9. Data Analysis and Statistical Procedures

Each experiment was repeated at least three times independently. Quantitative data were expressed as mean ± S.D. Statistical analysis was performed with an unpaired two-tailed Student’s *t*-test, and effects were considered statistically significant at * *p* < 0.05. One representative immunoblot of three independent experiments was shown in the results.

## 3. Results

### 3.1. FAM20B Gain-of-Function Reduces the Synthesis of PG–GAG Chains

**Figure 1:**
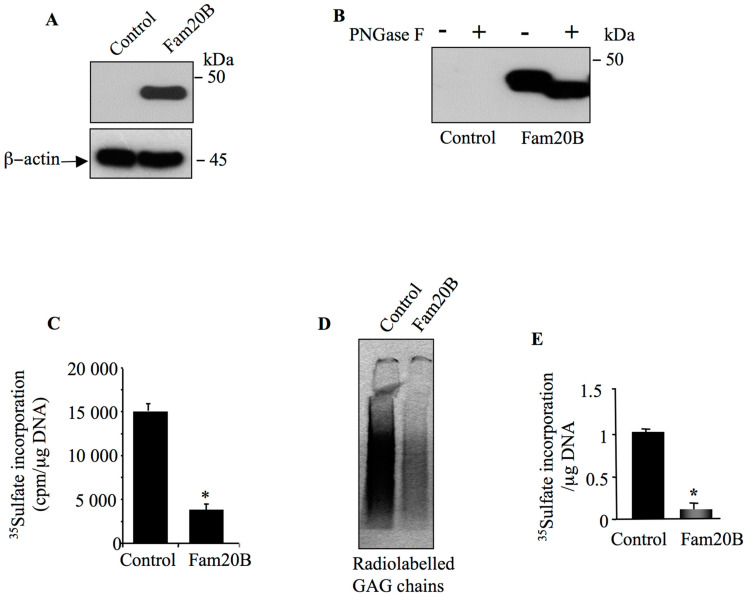
FAM20B blocks the synthesis of PG–GAG chains. HEK293 cells were transfected with pCMV-FAM20B or pCMV empty vector (control), and (**A**) FAM20B was detected by Western blot using anti-FAM20B antibodies. β-actin was used as loading control. (**B**) Western blot analysis of the sensitivity of FAM20B to digestion with PNGase F. (**C**) Analysis of the level of PG–GAG chain synthesis in HEK293 cells transfected with either FAM20B expression vector or empty plasmid (control) using ^35^S-sulfate incorporation method. (**D**) SDS-PAGE analysis of radiolabeled GAG chains produced in culture medium of HEK293 cells transfected with either FAM20B expression vector or empty plasmid (control) using ^35^S-sulfate incorporation method. The amounts of ^35^S-sulfate GAGs were normalized to DNA and relative to control. (**E**) The bar graph represents the quantification of GAG chains on the autoradiography of the SDS-PAGE. Data were presented as mean ± SD of three separate experiments. Statistical significance was evaluated using Student’s *t* test (*, *p* < 0.05).

It has been shown that loss of FAM20B impairs the synthesis of PGs leading to premature termination of PG–GAG chain elongation [25]. To gain more insight into the role of FAM20B in the synthesis of PG–GAG chains, we sought to determine whether *FAM20B* gain-of-function affects the synthesis of PG–GAG chains. To this end, we expressed FAM20B in HEK293 cells and analyzed the impact on the synthesis of PGs. Western blot analysis showed that cells transfected with the expression vector pCMV-FAM20B efficiently expressed a polypeptide of about 46 kDa corresponding to FAM20B protein, which was absent in cells transfected with pCMV empty vector (control) (Figure 1A). FAM20B contains potential N-glycosylation sites. To test whether FAM20B is N-glycosylated, proteins from HEK293 cells expressing FAM20B were treated with the enzyme N-glycosidase F (PNGase) and analyzed by Western blot. Treatment with PNGase produced a decrease of about 2 kDa in the apparent molecular mass of FAM20B polypeptide (Figure 1B), indicating that FAM20B is sensitive to PNGase and therefore is N-glycosylated. We next analyzed the effect of FAM20B gain-of-function on the synthesis of GAG chains using metabolic incorporation of radiolabeled [^35^S]-sulfate into GAG chains of PGs. Interestingly, the level of PG synthesis was decreased by 75% in cells transfected with FAM20B expression vector compared to control cells transfected with empty vector (Figure 1C). This was confirmed by SDS-PAGE analysis of radiollabeled GAG chains of PGs which shows strong reduction (90%) in overall GAG chains produced in FAM20B expressing cells compared to control (Figure 1D,E). These results indicated that FAM20B gain-of-function strongly reduced the synthesis of PG–GAG chains.

### 3.2. FAM20B Gain-of-Function Reduces the Synthesis of Both CS– and HS–GAG Chains

**Figure 2:**
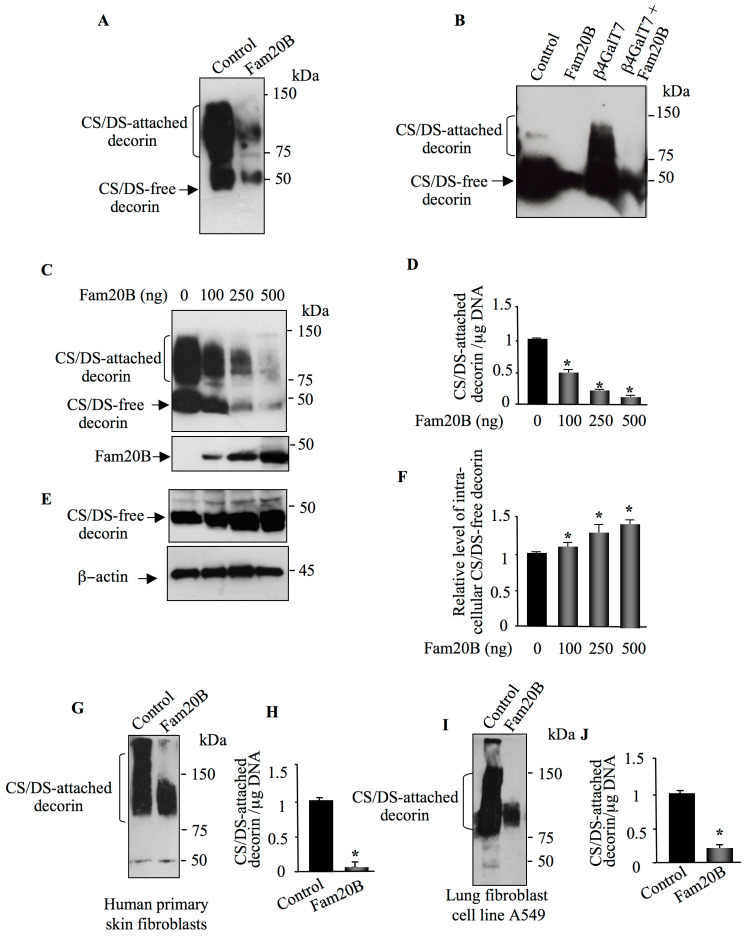
FAM20B suppression of GAG chain synthesis is dose-dependent. (**A**) HEK293 cells were co-transfected with pCMV-DCN along with pCMV-FAM20B or empty pCMV vector (control) and the expression of DCN was analyzed in medium by Western blot using anti-DCN antibodies. (**B**) CHO PgsB-618 cells were co-transfected with pCMV-DCN along with empty pCMV (control), pCMV-FAM20B, pCMV-β4GALT7 or pCMV-FAM20B, and pCMV-β4GALT7. DCN produced in the medium was analyzed by Western blot using anti-DCN antibodies. (**C**) Western blot analysis of FAM20B and DCN produced in the medium HEK293 cells transfected with pCMV-DCN and increased concentrations of FAM20B. (**D**) The bar graph represents the quantification of DCN in the Western blot. (**E**) Detection of intracellular DCN in cells transfected with pCMV-DCN and increased concentrations of FAM20B. β-actin was used as loading control. (**F**) The bar graph represents the quantification of DCN in the Western blot. (**G**) Western blot analysis of secreted DCN in the medium of human primary skin fibroblasts transfected with pCMV-FAM20B or empty vector (control). (**H**) The bar graph represents the quantification of DCN in the Western blot. (**I**) Detection of secreted DCN in the medium of A549 cells transfected with pCMV-FAM20B or empty vector (control). (**J**) The bar graph represents the quantification of DCN in the Western blot. Data were presented as mean ± SD of three separate experiments. Statistical significance was evaluated using Student’s *t* test (*, *p* < 0.05).

Given the fact that FAM20B phosphorylates the xylose residue of the tetrasaccharide linker common to both CS and HS, we hypothesized that FAM20B may impact the synthesis of both HSPGs and CSPGs. To examine this hypothesis, we used decorin and syndecan 4 as reporter for the synthesis of CSPGs and HSPGs, respectively. HEK293 cells were transfected with pCMV-FAM20B expression vector or pCMV empty vector (control) together with either pCMV-DCN vector expressing decorin or pCMV-HA-SDC4 vector expressing HA-syndecan 4 with an N-terminal tagged HA epitope. The expression of decorin and syndecan 4 was analyzed by Western blot. Transfection of HEK293 cells with pCMV-DCN vectors resulted, as expected, in the secretion in the medium of a high amount of CS/DS-attached decorin that migrates as an elongated smear during gel electrophoresis due to the heterogeneity of the attached GAG chains; however, when decorin was co-expressed with FAM20B, the amount of CS/DS-attached decorin in the medium was strongly reduced (Figure 2A). To further confirm that FAM20B reduces the synthesis of GAG-attached decorin, we took advantage of CHO pgs-B618 cells deficient for β4GalT7 enzyme. These cells initiate GAG synthesis by catalyzing the transfer of xylose residues on the PG core protein, but they are not able to catalyze the second step which consists of the addition of galactose on the xylose residue due to lack of β4GALT7, therefore precluding the formation of completed tetrasaccharide primer and subsequent elongation of the PG–GAG chain. Expression of decorin in CHO pgs-B618 cells resulted in the synthesis in the culture medium of a high amount of GAG chain-free decorin (Figure 2B). As expected, co-expression of decorin with β4GALT7 restored the synthesis of GAG-attached decorin. However, when decorin and β4GALT7 were expressed along with FAM20B, the synthesis of GAG-attached decorin was dramatically reduced (Figure 2B), indicating that *FAM20B* gain-of-function blocks GAG chain formation.

Next, we examine whether the effect of FAM20B on the synthesis of GAG-attached decorin is dose-dependent. To this end, HEK293 cells were transfected with decorin expression vector along with increasing concentrations of FAM20B expression vector. Remarkably, as shown in Figure 2C, the synthesis of GAG-attached decorin decreases with increased expression of FAM20B. Indeed, the amount of decorin expressed dropped by 50%, 70%, and 95% when cells were transfected with 100 ng, 250 ng, and 500 ng of FAM20B expression vector, respectively (Figure 2C,D). This data clearly indicated that inhibition of the synthesis of GAG-attached decorin by FAM20B is dose-dependent. Interestingly, analysis of intracellular decorin in cell lysates of control and FAM20B expressing cells clearly revealed the presence of a high amount of GAG chain-free decorin inside the cells expressing FAM20B, which increases with increasing expression of FAM20B (Figure 2E). The amount of intracellular GAG chain-free decorin was increased by about 10%, 30%, and 40% in cells transfected with 100 ng, 250 ng, and 500 ng of FAM20B expression vector, respectively, compared to control (Figure 2E,F). These results indicate that *FAM20B* gain-of-function led to accumulation of GAG chain-free decorin inside the cells which is not prone to secretion, due probably to the blockage in the elongation of the CS/DS GAG chain. To determine whether this process is cell type specific, we studied the effect of FAM20B expression on the synthesis of decorin in human primary skin fibroblasts and lung fibroblast cell line A549, which naturally produce and secrete a high amount of CS/DS-attached decorin. As expected, analysis of decorin from conditioned medium of human primary skin fibroblast cells showed a high amount of CS/DS-attached decorin, as revealed by immunoblotting using anti-decorin antibodies (Figure 2G); however, transfection with FAM20B expression vector led to a strong decrease (90%) in CS/DS-attached decorin in the medium (Figure 2G,H). Similar results were obtained in lung fibroblast cells A549. Transfection with FAM20B expression vector reduced the amount of GAG-attached decorin secreted in the medium by about 80%, compared to control (Figure 2I,J). Altogether, these results clearly showed that the effect of FAM20B on the synthesis of CS/DS-attached decorin is dose-dependent and is not cell-type specific.

**Figure 3:**
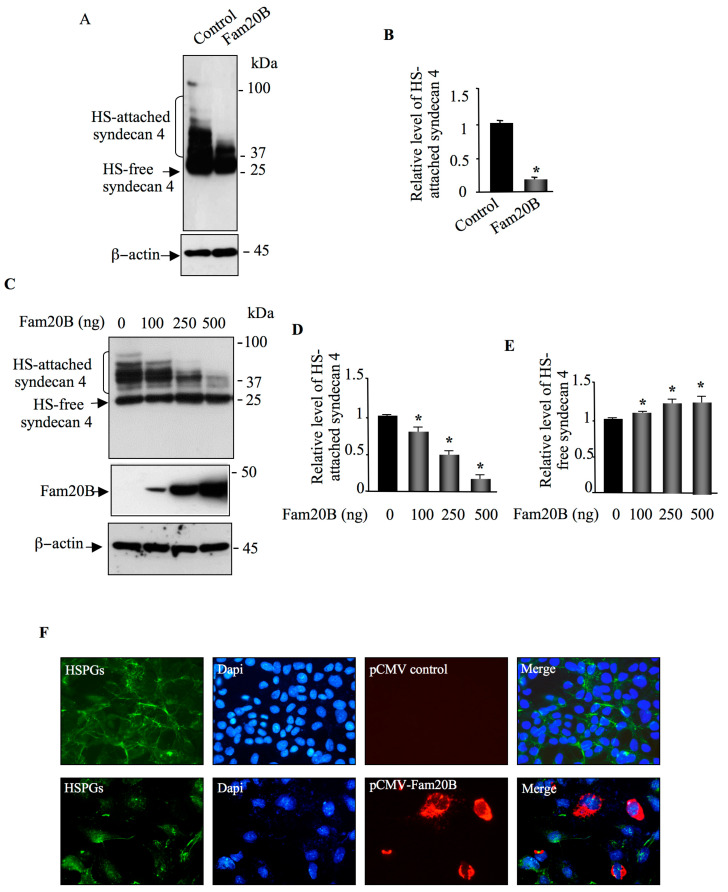
FAM20B blocks the synthesis of HSPGs. (**A**) HEK293 cells were co-transfected with pCMV-HA-SDC4 and either pCMV-FAM20B or empty pCMV (control) and SDC4 was analyzed in cell lysate by Western blot using anti-HA antibodies. β-actin was used as loading control. (**B**) The bar graph represents the quantification of the Western blots. (**C**) Western blot analysis of SDC4 and of FAM20B in cell lysate of HEK293 cells transfected with increased concentrations of FAM20B. β-actin was used as loading control. (**D**) The bar graph represents the quantification of HS-attached SDC4 in the Western blot. (**E**) The bar graph represents the quantification of HS-free SDC4 in the Western blot. (**F**) CHO-K1 cells were transfected with pCMV-FAM20B or empty pCMV (control) vector, and expression of cell surface HSPGs was examined by immunofluorescence using anti-heparan sulfate antibody 10E4 (green). The expression of FAM20B was analyzed using anti-FLAG antibody (red). Nuclei were stained (blue) using Hoechst/PBS solution. Digital images were captured with an inverted microscope, Leica DMI3000 (40×). Data were presented as mean ± SD of three separate experiments. Statistical significance was evaluated using Student’s *t* test (*, *p* < 0.05).

We next tested whether FAM20B produced similar inhibitory effect on the synthesis of HS-attached PGs, as observed for CSPG. For these purposes, we used syndecan 4 as HSPG reporter. HEK293 cells were transfected with HA-syndecan 4 expression vector and pCMV-empty vector (control) or with HA-syndecan 4 and FAM20B expression vectors. Transfection of cells with HA-syndecan 4 vector resulted in high expression of HS-attached syndecan 4 that migrates as a smear in SDS-PAGE (Figure 3A). Interestingly, co-expression with FAM20B led to a remarkable reduction (80%) in the synthesis of HS-attached syndecan 4 (Figure 3A,B), indicating that *FAM20B* gain-of-function dramatically decreases the synthesis of HS-attached syndecan 4. To determine whether reduction in the synthesis of HS-attached syndecan 4 is FAM20B dose-dependent, HEK293 cells were transfected with HA-syndecan 4 expression vector along with increasing concentrations of FAM20B expression vector. The results showed that the amount of HS-attached syndecan 4 dropped by about 20%, 50%, and 80% when cells were transfected with 100 ng, 250 ng, and 500 ng of FAM20B expression vector, respectively (Figure 3C,D). In contrast, the amount of HS-free syndecan 4 in the cells increased by about 10% to 25% with increased expression of FAM20B (Figure 3C,E). Altogether, these results demonstrated that gain-of-function of *FAM20B* reduced the synthesis of PGs of both types, CS and HS, and led to accumulation of GAG-free PG core protein inside the cells.

Cell surface HS-attached PGs are important for many vital cell-signaling processes [4,5,6]. To examine the effect of *FAM20B* gain-of-function on cell surface HSPGs, we carried out indirect immunofluorescence analysis of cell surface HSPGs in CHO-K1 cells transfected with FAM20B expression vector or with pCMV vector (control), using anti-HS monoclonal antibody 10E4, which is commonly used to detect HS chains of PGs [31,32,33]. Prominent staining of the cell membrane HSPGs was observed in CHO-K1 cells transfected with empty vector (control), whereas no or low HSPG staining could be observed in cell membrane of cells transfected with FAM20B expression vector (Figure 3F). When recombinant cells were probed with anti-FAM20B antibodies, efficient expression of FAM20B was revealed, whereas cells transfected with pCMV (control) showed no staining (Figure 3F). Noteworthy, when cells expressed FAM20B, no or very low staining for cell surface HSPG was observed (Figure 3F, Merge), indicating that *FAM20B* gain-of-function impaired the synthesis of cell surface HS–GAG chains.

### 3.3. XYLP Rescues the Synthesis of GAG-Attached PG Blockage Induced by FAM20B

**Figure 4:**
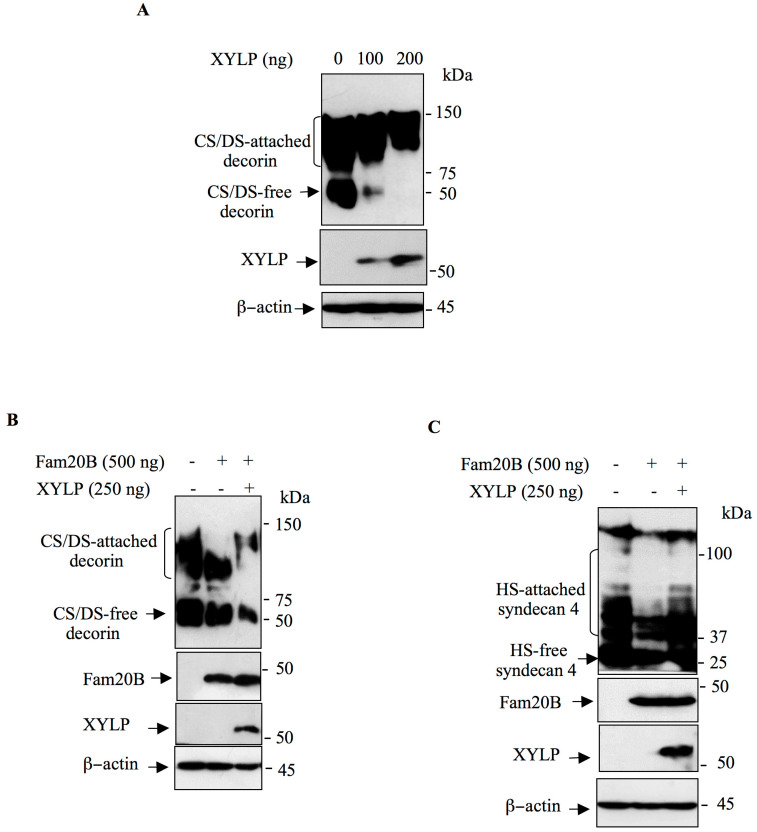
XYLP rescues the synthesis of GAG-chains produced by FAM20B. (**A**) HEK293 Cells were transfected with pCMV-DCN together with increased concentrations of pCMV-HA-XYLP. Expression of DCN and XYLP was analyzed by Western blot using anti-DCN and anti-HA antibodies, respectively. β-actin was used as loading control. (**B**) HEK293 cells were transfected with pCMV-DCN (control), pCMV-DCN and pCMV-FAM20B, or pCMV-DCN and pCMV-FAM20B together with pCMV-HA-XYLP. DCN, FAM20B, and XYLP were analyzed by Western blot using anti-DCN, anti-FAM20B, and anti-HA antibodies, respectively. β-actin was used as loading control. (**C**) HEK293 cells were transfected with pCMV-HA-SDC4, pCMV-HA-SDC4 and pCMV-FAM20B, or pCMV-HA-SDC4 and pCMV-FAM20B together with pCMV-FLAG-XYLP. SDC4, FAM20B, and XYLP were analyzed in cell lysate by Western blot using anti-HA, anti-FAM20B and anti-FLAG antibodies, respectively. β-actin was used as loading control. Data are presented as mean ± SD of three separate experiments.

It has been shown that XYLP is the 2-phosphoxylose phosphatase that de-phosphorylates xylose in the tetrasaccharide linker region of PGs [27]. We hypothesize that *FAM20B* gain-of-function may result in sustained phosphorylation of xylose residue which may lead to blockage in the elongation of CS and HS GAG chains; therefor expression of XYLP may rescue the elongation process. We first analyzed the effect of XYLP on the synthesis of decorin in HEK293 cells. Cells were transfected with pCMV-DCN and empty pCMV (control) vector or with pCMV-DCN and increased concentrations of pCMV-XYLP expression vector. As shown in Figure 4A, expression of XYLP increased, in a dose-dependent manner, the amount of GAG-attached decorin. Indeed, the amounts of CS/DS free decorin present in the medium gradually decreased as the concentration of XYLP increased (Figure 4A). This strongly suggest that *XYLP* gain-of-function enhanced the synthesis of GAG-attached decorin.

To determine whether expression of XYLP may rescue FAM20B-induced inhibition of GAG-attached decorin, HEK293 cells were transfected with decorin and FAM20B expression vector or with decorin and FAM20B along with XYLP expression vector. As expected, GAG-attached decorin is efficiently produced in cells transfected with decorin expression vector, whereas its expression was reduced in cells expressing FAM20B (Figure 4B). Remarkably, expression of XYLP rescued the synthesis of GAG-attached decorin leading to the synthesis of high molecular mass CS/DS attached decorin, therefore overcoming the inhibitory effect of FAM20B on the synthesis of CS/DS attached decorin (Figure 4B). Similar results were obtained for syndecan 4. Transfection of HEK293 cells with HA-syndecan 4 expression vector resulted in the synthesis of a high amount of GAG-attached syndecan 4; however co-expression with FAM20B dramatically reduced the synthesis of HS-attached syndecan 4 (Figure 4C). Interestingly, expression of XYLP rescued FAM20B-induced inhibition of the synthesis of HS-attached syndecan 4 (Figure 4C). Altogether, these results demonstrate that *FAM20B* gain-of-function blocks the synthesis of GAG-attached PGs that can be rescued by XYLP.

### 3.4. Aspartic Acid Residues in Catalytic Domain and DFG Motif Are Essential for FAM20B Activity

**Figure 5:**
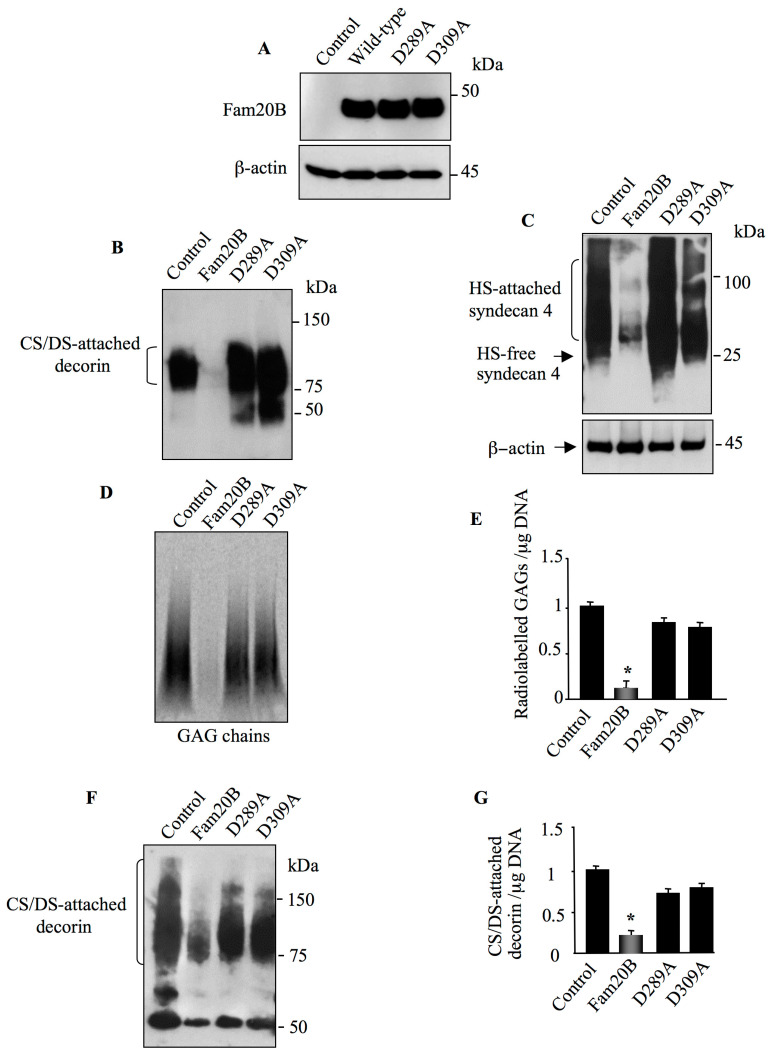
The aspartic acid residues Asp^289^ and Asp^309^ are essential for FAM20B function. (**A**) Detection of wild-type FAM20B and mutants in HEK293 cells transfected with pCMV-FAM20B, pCMV-FAM20B^D289A^, pCMV-FAM20B^D309A^ or pCMV empty vector (control) using anti-FAM20B antibodies. β-actin was used as loading control. (**B**) HEK293 cells were co-transfected with pCMV-DCN, along with pCMV-FAM20B, pCMV-FAM20B^D289A^, pCMV-FAM20B^D309A^ or pCMV empty vector (control) while DCN produced in the medium was analyzed by Western blot using anti-DCN antibodies. (**C**) HEK293 cells were co-transfected with pCMV-HA-SDC4 along with pCMV-FAM20B, pCMV-FAM20B^D289A^, pCMV-FAM20B^D309A^ or pCMV empty vector (control) while SDC4 was analyzed by Western blot using anti-HA antibodies. β-actin was used as loading control. (**D**) PG–GAG chains produced in the medium of HEK293 cells transfected with pCMV-FAM20B, pCMV-FAM20B^D289A^, pCMV-FAM20B^D309A^ or pCMV empty vector (control) were metabolically labelled by ^35^S-sulfate incorporation and isolated by CPC precipitation, then separated by SDS-PAGE, and revealed by autoradiography. (**E**) The bar graph represents the quantification of ^35^S-sullfate radiolabeled GAG chains of the autoradiography. (**F**) A549 cells were transfected with pCMV-FAM20B, pCMV-FAM20B^D289A^, pCMV-FAM20B^D309A^ or pCMV empty vector (control) while DCN secreted by the cells in the medium was analyzed by Western blot using anti-DCN antibodies. (**G**) The bar graph represents the quantification of the Western blot. Data are presented as mean ± SD of three separate experiments. Statistical significance was evaluated using Student’s *t* test (*, *p* < 0.05).

Based on the structure/function studies and 3D structure analysis of *C. elegans* FAM20C (*ce*FAM20C), it has been shown that the aspartic residue of the ^387^DHG^389^ sequence, a variant of the DFG motif present in canonical protein kinases which co-ordinates Mn^2+^ cation, as well as the putative catalytic aspartic residue present in the catalytic segment ^366^DRHHYE^371^ are essential for ceFAM20C activity [23]. The corresponding aspartic residues are present in the human FAM20B and are Asp^309^ and Asp^289^, respectively. We therefore explored the effect of the mutation of these residues on human FAM20B activity. To this end, Asp^309^ to alanine (D309A) and Asp^289^ to alanine (D289A) mutants of FAM20B were engineered and expressed in CHO-K1 cells. Western blot analysis showed that FAM20B^D309A^ and FAM20B^D289A^ mutants were expressed in a similar amount as wild-type FAM20B protein (Figure 5A). To determine whether the D309A and D289A mutations impair FAM20B activity, decorin and syndecan 4 were co-expressed with either wild-type FAM20B or with the mutants FAM20B^D309A^ and FAM20B^D289A^, respectively, and GAG-attached decorin and syndecan 4 were analyzed by immunoblot. In contrast to wild-type FAM20B, co-expression with the mutants FAM20B^D309A^ and FAM20B^D289A^ did not suppress the synthesis of either CS/DS-attached decorin (Figure 5B) nor that of HS-attached syndecan 4 (Figure 5C). Indeed, a high amount of CS/DS-containing decorin and HS-attached syndecan 4 was produced in the presence of FAM20B^D309A^ and FAM20B^D289A^, respectively, indicating that mutation of Asp^309^ and Asp^289^ to alanine abolished the ability of FAM20B to suppress PG–GAG synthesis and therefore to impair the activity of FAM20B. To further confirm this finding, wild-type FAM20B and the mutants FAM20B^D289A^ and FAM20B^D309A^ were transfected into HEK293 cells and neosynthesized PG–GAG chains were radiolabeled using ^35^S-sulfate incorporation. SDS-PAGE analysis of radiolabeled GAG chains produced by FAM20B^D289A^ and FAM20B^D309A^ expressing cells showed a similar pattern as in the control (Figure 5D), whereas cell expressing wild-type FAM20B presented strong reduction (90%) in overall GAG chains produced (Figure 5D,E).

We next analyzed the effect of FAM20B mutations on the synthesis of decorin in human lung cancer cell line A549, which abundantly produced this PG in the medium. Immunoblot analysis of decorin from culture supernatants of lung fibroblast cells expressing FAM20B^D289A^ and FAM20B^D309A^ mutants showed a high amount of CS/DS-attached decorin as in control cells (Figure 5F). In contrast, the amount of CS/DS-attached decorin produced by cells expressing wild-type FAM20B was decreased by about 75% (Figure 5F,G). These results showed that FAM20B^D289A^ and FAM20B^D309A^ mutants did not suppress the synthesis of CS/DS-attached decorin in lung fibroblasts as occurs with wild-type FAM20B. Thus, these results clearly demonstrated that expression of an altered form of FAM20B did not alter the synthesis of GAG-attached PGs, therefore confirming that *FAM20B* gain-of-function is a negative regulator of PG synthesis.

### 3.5. Knockout of FAM20B Reduced the Synthesis of Both CS and HSPGs

**Figure 6:**
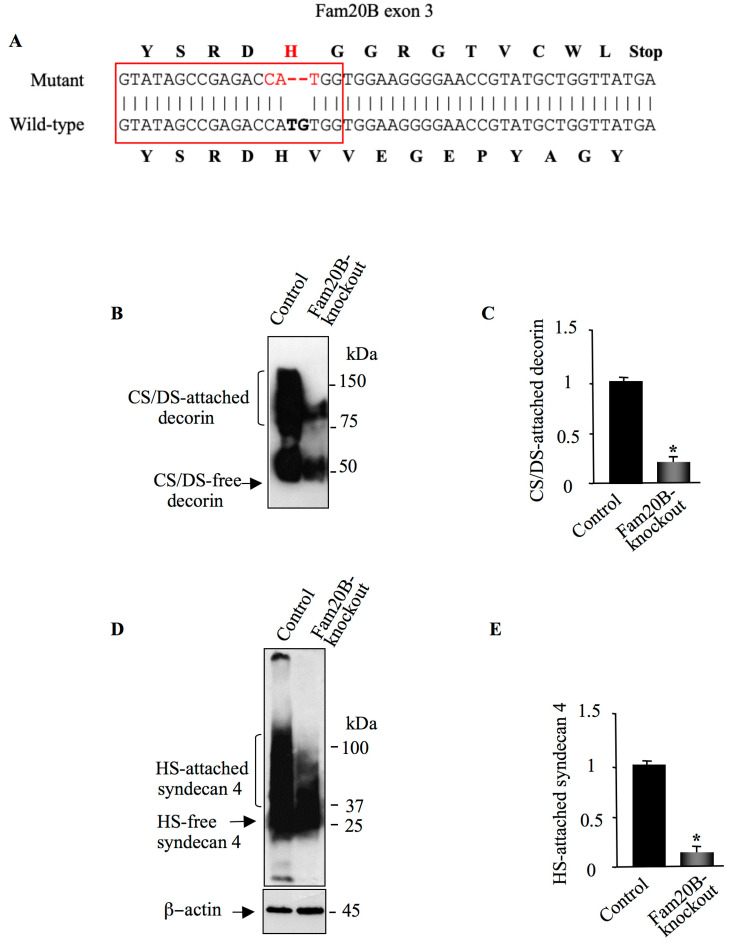
CRISPR/Cas9 knockdown of FAM20B. (**A**) Alignment of FAM20B targeted sequence from wild-type and mutant HEK293 cells. Red box represents RNAg sequence. (**B**). Detection of DCN in the culture medium of control and FAM20B-knockout HEK293 cells transfected with pCMV-DCN. (**C**) The bar graph represents the quantification of the Western blot. (**D**) Western blot analysis of SDC4 in cell lysate of HEK293 cells (control) and FAM20B-knockout HEK293 cells transfected with pCMV-HA-SDC4. β-actin was used as loading control. (**E**) The bar graph represents the quantification of the Western blot. Data are presented as mean ± SD of three separate experiments. Statistical significance was evaluated using Student’s *t* test (*, *p* < 0.05).

We next analyzed the effect of the loss of *FAM20B* in HEK293 cells on the synthesis of PG–GAG chains by generating *FAM20B*-knockout HEK293 cells using CRISPR/Cas9 technique. *FAM20B*-knockout clone harboring deletion mutation in exon 3 leading to premature stop codon was selected (Figure 6A) and transfected with vectors expressing either decorin or HA-syndecan 4. As shown by Western blot, transfection of control and *FAM20B*-knockout cells with decorin expression vectors resulted in the synthesis of a high amount of CS/DS-attached decorin (Figure 6B). However, the amount of GAG-attached decorin produced in *FAM20B*-knockout HEK293 cells was reduced by about 75%, compared to control cells (Figure 6B,C). Similar results were observed for HSPG syndecan 4. Expression of HS-attached syndecan 4 was dramatically reduced in *FAM20B*-knockout cells, compared to control cells. (Figure 6D,E). These results showed that absence of *FAM20B* is associated with decreased synthesis of GAG-attached PGs. Altogether, these data showed that both loss- and gain-of-function of *FAM20B* downregulate the synthesis of GAG-attached PGs arguing for a strict dependence of PG synthesis on FAM20B expression levels and therefore suggest that modulation of the expression of FAM20B regulates the rate of PG synthesis.

### 3.6. FAM20B Inhibits Proliferation and Migration of Glioblastoma Cells

**Figure 7:**
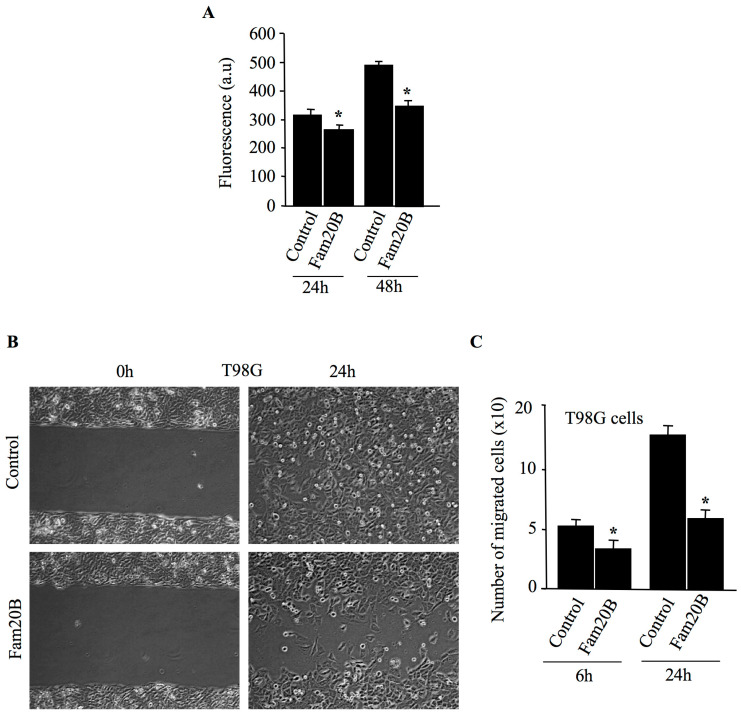
FAM20B reduces the proliferation and migration of glioblastoma cells T98G. (**A**) Glioblastoma cells T98G were plated in 96 well plates and transfected with pCMV-FAM20B or empty pCMV vector (control), while cell proliferation was measured by CyQUANT NF Cell Proliferation Assay Kit at 24 h and 48 h after transfection. (**B**) Migration of glioblastoma cell line T98G transfected with pCMV-FAM20B or pCMV empty vector (control) was assessed using scratch wound-healing assay as described in the Materials and Methods section and cell migration was photographed at 0 and 24 h after scratch by phase-contrast microscopy (×10). (**C**) The bar graph depicts the number of cells that migrate in the scratch area after 6 h and 24 h. Data are presented as mean ± SD of three separate experiments with three replicates each. Statistical significance was evaluated using Student’s *t* test (*, *p* < 0.05).

Because PGs play an important role in cell migration and proliferation, we hypothesized that FAM20B-dependent inhibition of PG–GAG synthesis may affect these processes. To test this hypothesis, FAM20B was expressed in brain tumor glioblastoma cell line T98G. Cell proliferation was measured at 24 h and 48 h after transfection. As shown in Figure 7A, glioblastoma cells transfected with FAM20B presented a reduction in cell proliferation of about 14% at 24 h and 28% at 48 h, compared to control. We next analyzed the ability of FAM20B-transfected cells to migrate using scratch wound-healing experiments. Interestingly, T98G cells transfected with FAM20B expression vector were impaired in their ability to migrate when compared to control cells (Figure 7B,C). The migration assays revealed a slower rate of cell migration by the FAM20B-transfected cells compared with control cells. Indeed, expression of FAM20B in glioblastoma cells reduced the number of cells that migrate in the scratch area by about 36% at 6 h and 53% at 24 h, compared to control (Figure 7B,C). These results support that FAM20B-dependant inhibition of PG–GAG synthesis impairs cell proliferation and migration, therefore highlighting the critical role of GAG chains of PGs in glioblastoma cell tumorigenesis.

## 4. Discussion

In this study we showed that *FAM20B* gain-of-function induced a strong reduction in overall PG–GAG chains produced in the cell. By using decorin and syndecan 4 as reporter proteins for CS and HS-attached PGs, we showed that *FAM20B* gain-of-function negatively regulates the synthesis of both CS- and HS-attached PGs. This was further confirmed using human skin and lung fibroblasts expressing a high amount of endogenous decorin in the medium. *FAM20B* gain-of-function blocks the synthesis of decorin in both skin and lung fibroblast cells. Similar results were obtained for endogenous HSPGs. By using anti-HS 10E4 antibody which is commonly used to detect HS chains of PGs [31,32,33], we showed that *FAM20B* gain-of-function strongly attenuates the expression of cell surface HSPGs. Remarkably, we showed that *FAM20B* gain-of-function reduced the synthesis of both CS/DS-attached decorin and HS-attached syndecan 4 in a dose dependent manner and induces intracellular accumulation of GAG-free decorin and syndecan 4. Therefore, this suggests that lack of GAG elongation prevents PGs from being secreted. Interestingly, reduced secretion in culture medium of decorin and syndecan 4 in FAM20B expressing cells was accompanied with increased amounts of intracellular non-elongated decorin and syndecan 4, which are probably not competent for secretion and accumulate in the cells. Altogether, these data clearly demonstrate that *FAM20B* gain-of-function negatively regulates the synthesis of PGs of both types, CS and HS. As the synthesis of CS and HS GAGs share a common tetrasaccharide primer, *FAM20B*, gain-of-function affects the synthesis of the primer. Indeed, blocking of any step in the synthesis of this primer will obviously impact the synthesis of both CS and HS.

Of note, it has been reported that stable expression of FAM20B in Hela cells increased the amounts of GAGs with particularly augmented short CS chains [24]. On the other hand, it has been shown that loss-of-function of XYLP, the 2-phosphoxylose phosphatase that de-phosphorylates xylose of PG primer region, decreased HS and CS synthesis, and inversely, gain-of-function increased their synthesis [27]. Using transient expression of FAM20B in several cell lines as well as primary fibroblast cells, we found that *FAM20B* gain-of-function reduced the synthesis of GAG-attached PGs in all the cells tested. The discrepancy may be due to the selection of stable clones or to an unknown factor that has to be identified. On the other hand, in agreement with our study, it was shown that the knockout of FAM20B in bone osteosarcoma cells, U2OS, led to reduction in the synthesis of GAGs [25]. This study also showed that GALTII presents higher activity towards phosphorylated gal-xylose, compared to its unphosphorylated counterpart; thus, loss of FAM20B causes impaired GALTII activity resulting in the formation of incomplete linkage tetrasaccharides, capped with sialic acid and cannot be elongated [25].

During the physiological conditions, there is a balance between the phosphorylation and dephosphorylation of the xylose by FAM20B and XYLP, respectively. Therefore, gain- or loss-of-function of either *FAM20B* or *XYLP* may disturb this balance, thus affecting the rate of GAG synthesis. How FAM20B inhibits the synthesis of GAG chains is unknown; however, one can hypothesize that *FAM20B* gain-of-function may lead to sustained phosphorylation of xylose residue on the core protein, therefore competing with XYLP phosphatase that de-phosphorylates xylose of PG linker region, a process that may be required prior to subsequent GAG elongation. Noteworthy, a working model for the role of FAM20B in the synthesis of the tetrasaccharide linkage and subsequent GAG elongation suggests that xylose phosphorylation by FAM20B occurs predominantly after addition of the first galactosyl residue to the growing linkage region, and that the removal of the xylose 2-phosphate moiety by the xylose 2-phosphatase, XYLP, occurs following completion of the tetrasaccharide linkage to allow GAG elongation [34]. If the phosphate is not removed by XYLP, EXTL2 transfers the GlcNAc residue via an α1,4-linkage on the phosphorylated tetrasaccharide, leading to synthesis of a phosphorylated pentasaccharide structure, unable to serve as primer for CS/HS polymerization [27]. Whereas, it has been reported that phosphorylated tetrasaccharide is a preferred substrate for ChGn-1 enzyme that transfers a GalNAc residue to the phosphorylated tetrasaccharide in the protein linkage region of CS.

Interestingly, it has been shown that phosphorylation of xylose before addition of the first galactosyl residue occurs. Indeed, analysis of [^32^P]-labelled O-linked glycan chains and stubs attached to intracellular decorin, revealed that 52% of xyl-decorin was phosphorylated, therefore indicating that phosphorylation of xylose occurs before addition of galactose residue on xyl-decorin, continues when the gal residues are added, and is complete at the trisaccharide stage [34,35]. In a previous study, we reported that β4GALT7 efficiently catalyzes the transfer of galactose residue onto the non-phosphorylated xyloside analog, whereas the phosphorylated xylose analog at position 2-O was not substrate, suggesting that the 2-O phosphorylation precludes the transfer of the galactose on xylose [13]. In line with this, Siegbahn et al., showed that most modifications of position 2 in xylose, rendered analogs less prone to galactosylation by β4GALT7 [36], and showed that xylose analogs carrying modification on the 2-O position were not able to prime GAG synthesis in human cell lines [37]. These data indicate that the hydroxyl in position 2 might act as a hydrogen bond acceptor. The crystal structure of the β4GALT7 was published recently and revealed that the Tyr^177^, Tyr^179^, Trp^207^, and Leu^209^ are important for the hydrophobic binding of the xylose and that the Asp^211^ forms a strong hydrogen bond with the OH present in the 4th position of the acceptor xylose. Whereas, the Asp^212^ interacts via a hydrogen bond with the OH present at 2nd position of the xylose [38]. This suggested that phosphorylation of xylose, before addition of gal residue by FAM20B, may lead to premature termination of the tetrasaccharide primer synthesis and subsequently to unelongated GAGs. This may constitute a mechanism to counteract excessive synthesis of elongated GAG chains.

All the members of the FAM20 family contains a conserved C-terminal domain including the catalytic domain DRHHYE and a DFG motif which is crucial for metal ion binding. It has been shown that mutation of the aspartic acid residues in the catalytic segment and in the DFG motif of FAM20C abolished enzyme activity [21]. These aspartic acid residues are conserved in all members of the FAM20 family and correspond to Asp^289^ and Asp^309^ of the human FAM20B. Mutation of either of the two aspartic residues to alanine abolished the ability of FAM20B to block the synthesis of both CS- and HS-attached PGs, suggesting that mutation of Asp^289^ and Asp^309^ impaired FAM20B activity, consistent with the putative role of theses residues in catalysis and in the binding of the divalent cation Mn^2+^, respectively.

On the other hand, analysis of biological processes, including cell proliferation and migration indicated that both cell proliferation and migration were impaired in FAM20B-transfected cells. Expression of FAM20B in glioblastoma cells led to a significant reduction in proliferation and in the ability of cells to migrate in scratch-wound healing assays. The importance of PGs and their GAG chains in cell proliferation and migration is well established [39], and numerous PGs have been implicated in tumor growth and metastasis [40]. Therefore, FAM20B-dependent inhibition of PG–GAG synthesis could contribute to the cell proliferation and migration defects observed in glioblastoma cells.

## 5. Conclusions

Finally, our study showed that *FAM20B* gain-of-function led to blockage in the synthesis of GAG chains, whereas that of XYLP increased the synthesis thus providing novel insights into the role of FAM20B and XYLP in the synthesis of GAG chains of PGs. This strongly suggests that a balance between phosphorylation and dephosphorylation of the xylose by FAM20B and XYLP, respectively, is probably an essential factor for the regulation of the rate of PG synthesis. Therefore, studying the regulation of the expression of *FAM20B* and *XYLP* genes in normal and pathological conditions may help to understand the defects in the synthesis of GAG chains associated with several diseases such as cancer, atherosclerosis, and osteoarthritis. Several PGs are overexpressed in glioblastoma promoting aberrant activation of RTK signaling, which is associated with tumor progression and proliferation. Our findings on FAM20B inhibitory effects on proliferation and migration of glioblastoma cells provide evidence that GAG chains play a critical role in glioblastoma tumorigenesis and could lead to the development of new strategies against glioblastoma tumorigenesis.

## Data Availability

Data available on request.
